# Blueprints for healing: central nervous system regeneration in zebrafish and neonatal mice

**DOI:** 10.1186/s12915-025-02203-0

**Published:** 2025-04-30

**Authors:** Brianna R. Cellini, Sreeparvathy Vayankara Edachola, Timothy D. Faw, Valentina Cigliola

**Affiliations:** 1 Department of Psychology and Neuroscience, Duke University, Durham, NC 27710 USA; 2 Université Côte d’Azur, CNRS, Inserm, iBV, Nice, France; 3 Department of Orthopaedic Surgery, Duke University, Durham, NC 27710 USA; 4 Duke Institute for Brain Sciences, Duke University, Durham, NC 27710 USA; 5 Department of Pharmacology, Vanderbilt University, Nashville, TN 37232 USA; 6 Vanderbilt Brain Institute, Vanderbilt University, Nashville, TN 37232 USA

**Keywords:** Spinal cord, Brain, Injury, Regeneration, Zebrafish, Neonatal mice

## Abstract

In adult mammals, including humans, neurons and axons in the brain and spinal cord are inherently incapable of regenerating after injury. Studies of animals with innate capacity for regeneration are providing valuable insights into the mechanisms driving tissue healing. The aim of this review is to summarize recent data on regeneration mechanisms in the brain and spinal cord of zebrafish and neonatal mice. We infer that elucidating these mechanisms and understanding how and why they are lost in adult mammals will contribute to the development of strategies to promote central nervous system regeneration.

## CNS injury, current study models of regeneration and therapeutic approaches

Injuries to the central nervous system (CNS) are leading causes of long-term disability and can result in high costs of care [1]. Conditions such as stroke, traumatic brain injury (TBI), and spinal cord injury (SCI) account for over 60% of paralysis cases in the USA [2]. These injuries involve primary events, including neuronal death, axonal disruption and degeneration, and loss of synaptic connections. Secondary events often follow, characterized by infiltration of inflammatory cells from both central and peripheral sources [3]. Additionally, reactive astrocytes, pericytes, infiltrating fibroblasts, and Schwann cells gather at the lesion site, ultimately contributing to the formation of glial and fibrotic scars [4–9]. As these processes unfold, debris accumulation at the lesion site and the release of pro-inflammatory cytokines sustain inflammation, exacerbate cell death, and lead to partial or complete loss of function [3, 8]. In humans and other adult mammals, recovery after brain or spinal cord damage is limited. Lost neurons cannot be replaced, and scar formation is thought to create both physical and chemical barriers that impede axon regrowth upon trauma [10–12]. As a result, CNS injuries often lead to permanent impairments, including altered consciousness, cognitive dysfunctions, and deficits in autonomic, motor, and sensory function, causing significant disability and reduced quality of life.

To overcome the limited regenerative capacity of the adult CNS, scientists are attempting to harness the potential of cell transplantation in the context of CNS injury, largely based on the initial observation that fetal tissue transplants into the spinal cord can extend the critical period for developmental axon plasticity in rats [13]. As a result, clinical developments involving the use of various cell types, the scope of which is reviewed in detail elsewhere, are making progress toward clinical interventions [14, 15]. Among the most promising are neural progenitor cells (NPCs) that have been transplanted into the injured nervous system of mice and rats with mixed success in SCI and TBI models [14, 16]. These NPCs differentiated into glial cells and neurons integrating into the lesion site and extending axons [17–23]. The newly formed neurons establish connections with brain and brainstem projections, restoring some electrophysiological connectivity across the injury site. However, stem cell-based therapies are still in the preliminary stages, and the risks involved are not yet fully understood [17, 19–21].

Beyond regenerative approaches, recent studies have highlighted the potential of spinal cord electrical stimulation to restore neuronal function and walking following SCI in mouse models as well as human patients [24–31]. Similarly, non-invasive therapies, such as transcranial magnetic stimulation [32–35] and hyperbaric oxygen therapy [36], have helped restore motor and cognitive deficits following brain injury in humans. Despite the remarkable clinical potential of these strategies, they do not restore tissue integrity and, at best, result in partial functional recovery. Furthermore, a limited number of patients have received these interventions, long-term effects are unresolved, and the specific mechanisms and contributions from regions targeted by these procedures are still not entirely characterized [37, 38].

In the past few decades, a variety of animal models with remarkable regenerative abilities in the CNS have been identified. These include amphibians (newt, axolotl, frogs) [39–43], certain reptiles [44, 45], lampreys [46], eel [47], carp [48, 49], goldfish [50, 51], zebrafish [52, 53], and birds [54]. Among mammals, CNS regeneration has been observed in mice *(Mus)*, rat, and opossum at the neonatal stage [55–58] as well as in spiny mice *(Acomys)* which have emerged as an exciting organism for regenerative biology despite their currently limited commercial availability [59].

**Fig. 1:**
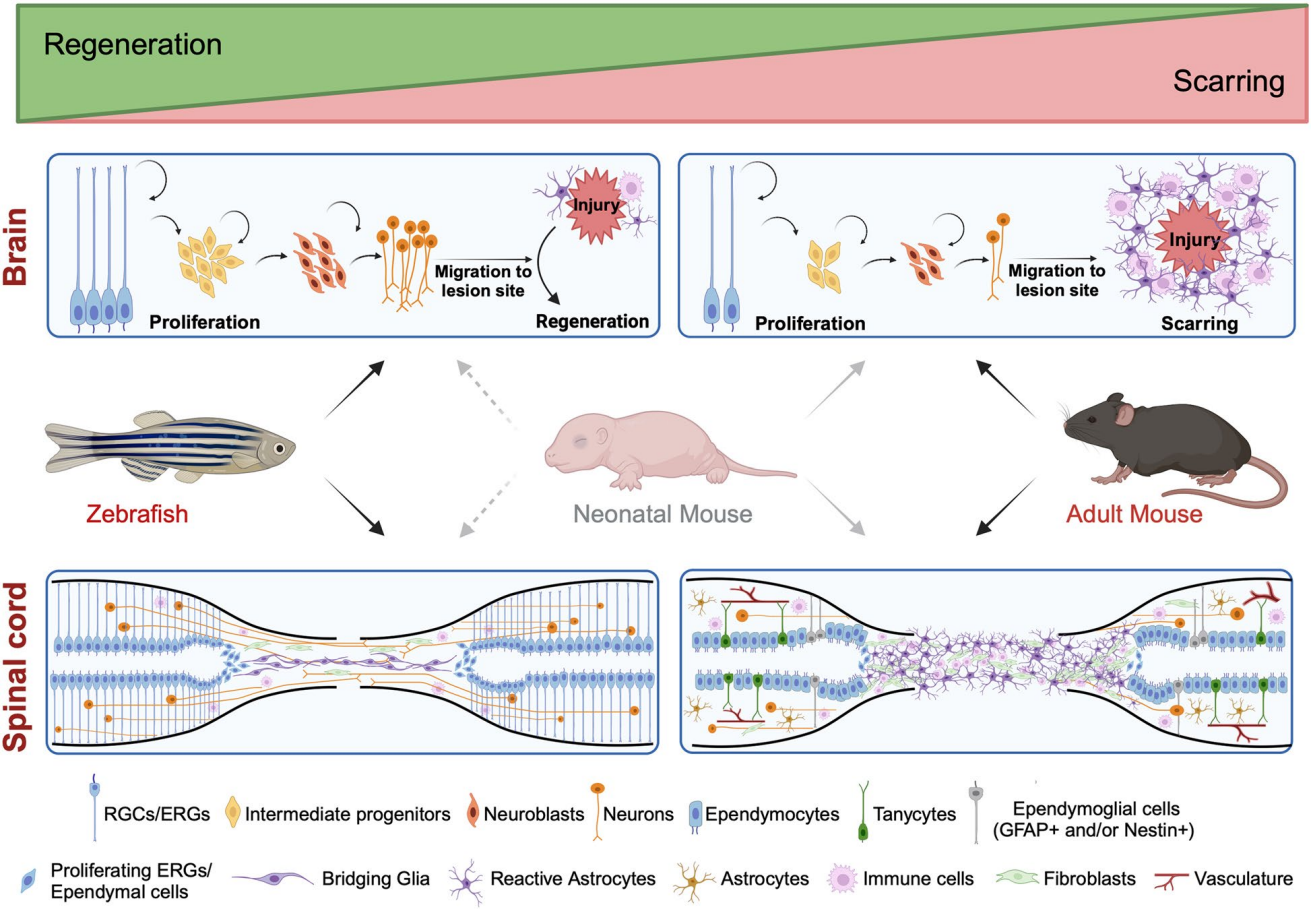
Regenerative potential of zebrafish and mouse central nervous system. *Top*, representation of the outcomes of brain injury in zebrafish, neonatal and adult mice. In zebrafish, injury is followed by proliferation of radial-glial cells (RGCs), differentiation, neurogenesis, and migration of newly formed neurons to the lesion site. Adult mammals are incapable of this regenerative response and undergo glial and fibrotic scarring at the injury site. Some regeneration is possible in neonatal mice. *Bottom*, spinal cord injury in zebrafish is also followed by extensive neurogenesis from ependymo radial-glial progenitors (ERGs) around the central canal, glial bridging, and axon regeneration, ultimately resulting in functional recovery. By contrast, in adult mammals, SCI leads to the formation of glial and fibrotic scarring that impedes regeneration and axonal growth across the lesion site, despite some compensatory axonal sprouting. Similar to zebrafish, neonatal mice are capable of axon regeneration after SCI. Figure generated using Biorender

While species like amphibians, reptiles, lampreys, and spiny mice have impressive regenerative abilities, they present challenges for scientific endeavor that include lack of broad availability, long generation times, and relative difficulty of genetic manipulation. These limitations complicate comparative biological, targeted gene-editing, or large-scale studies using modern molecular techniques such as CRISPR-Cas9, RNA interference, or transgenesis. In this review, we focus on CNS regeneration in zebrafish and neonatal mice, two well established models that are easily accessible, compatible with various genetic tools, and share similar regenerative mechanisms (Fig. 1). We highlight mechanisms of natural regeneration in these species while comparing them to the injury responses observed in adult mice, an approach that we believe can advance basic science and inspire new CNS treatments (Fig. 1).

## Zebrafish and neonatal mice as models for innate CNS regeneration

Zebrafish have emerged as a powerful model for studying CNS regeneration due to their unique regenerative abilities and advantages, including the production of large clutches and rapid development. Experimental manipulations such as transgenesis, targeted gene knockout, and genome-editing knock-ins are well-established, aided by a fully sequenced and annotated genome. Additionally, protocols for cell-specific manipulations and knock-in/conditional alleles are available, although their application is still developing [60]. Larval zebrafish are useful due to their transparency and rapid regeneration, while adults allow the study of more complex behaviors. Importantly, CNS regeneration mechanisms are mostly conserved between larval and adult stages, though some pro-regenerative tissues or cell types may not be fully developed in larvae. For example, functional and integrated adaptive immune response is not present until 3 weeks post-fertilization [61]. This implies that some differences in regeneration mechanisms may still exist and are yet to be discovered. Certain considerations must be made in using zebrafish as model systems to study CNS regeneration. The cellular composition of the zebrafish brain and spinal cord differs for certain aspects from those of mammalian tissues. Additionally, restoration of functional potential, such as swim capacity after SCI, is not always full [52]. Lastly, despite remarkable discoveries, gaps still exist regarding the metabolic effects of regeneration, fibrosis, the effect of physical or mechanical stress, and the mechanisms of growth control. As discoveries on CNS regeneration in zebrafish advance, these limitations will help shape key questions for future research.

Neonatal mice have also emerged as a valuable model to study CNS regeneration, offering a range of tools such as optogenetics, chemogenetics, and genetic manipulation techniques, such as knockout and inducible transgenic systems. Additionally, comparing regeneration in neonatal versus adult mice provides important insights into age-related differences in regenerative capacity. Neonatal mice can regenerate during a transient window of approximately 1 week after birth, and this regenerative capacity declines as they age [62]. Age dependency raises the question as to whether regenerative responses are relevant in adult cell populations. There has been discussion on whether tissue renewal and growth after an injury at early postnatal stages represent a continuation of embryonic development or whether injuries to neonatal tissues more readily reactivate developmental pathways that promote regeneration [62]. Making a clear distinction between regeneration and developmental growth is experimentally challenging. However, research in zebrafish indicates that larval spinal cord regeneration does not involve a continuation or reactivation of developmental programs. Instead, it relies on injury-specific responses [63]. This finding invites consideration of whether the same applies to neonatal mice.

## Guiding questions to study innate CNS regeneration

The discovery that both the zebrafish and neonatal mouse CNS can spontaneously regenerate after a variety of injury methods (Table 1) prompts numerous questions. For example: What are the signals that trigger, control, and restrict the repair process? Are these signals shared across these two species? Do they result from cues released by dying cells or are they part of more general responses to damage, like the generation of reactive oxygen species or inflammatory processes? Lastly, a crucial question revolves around why the neonatal CNS allows for regeneration and what inhibitory mechanisms exist in the adult CNS that hinder cell replacement after injury. In the sections below, we synthesize our current understanding of the cellular, molecular, genetic, and functional mechanisms of brain and spinal cord regeneration. We focus on processes that are conserved in zebrafish, at larval and adult stages, and in neonatal mice, while comparing to responses in adult mice (Fig. 1). We recognize this approach captures a specific portion of the broad efforts in the field, and we reference other reviews to overcome coverage limitations.

**Table 1:** Most common injury methods used to study brain and spinal cord regeneration in zebrafish and neonatal mice. *, authors employed a stab wound injury model to completely transect the larval spinal cord. References not discussed in the text are indicated in italics

	**Method**	**Pros**	**Cons**	**Organ**	**References**
**Zebrafish**	Crush/ compression	• Clinically relevant	• High variability	Brain	N/A
				SC	[64–67] (Adults)
	Stab wound	• Localized damage • Technically simple	• Injury depth difficult to control	Brain	[68–73] (Adults)
				SC	*Refer to larval transection studies
	Transection	• Localized lesion • Complete axon cut • Consistent, reliable	• Most severe • Infection risk • Low viability • Rarely encountered clinically	Brain	N/A
				SC	[74–83] (Larvae), [52, 75, 77, 84–102] (Adults)
	Laser ablation	• High reproducibility • High accuracy • Non-invasive	• Needs advanced microscopy equipment	Brain	[103] (Larvae), [104] (Juveniles)
				SC	[105]* (Larvae)*
	Chemogenetic ablation	• High spatial and cell type specificity • Possibility of temporal control	• Off target effects • Incomplete / variable ablation • Toxicity of administered drugs	Brain	[106, 107]
				SC	[108, 109] (Larvae) [110] (Adult)
	Excitotoxic	• Targeted cell ablation • Consistent and reproducible damage	• Possibility of off target effects • Systemic toxicity	Brain	[111]* (Adult)*
				SC	N/A
	Electroablation	• High reproducibility • Precise spatial control • Rapid and reproducible • Minimal systemic effects	• Technically demanding • Limited cell type specificity • Limited to accessible tissues	Brain	N/A
				SC	[112] (Larvae)
**Neonatal mouse**	Crush	• High clinical relevance • Possible to control injuryextent (complete vs.incomplete)	• High variability when performing incompleteinjuries • Damage to surrounding tissues	Brain	N/A
				SC	[113, 114]
	Stab wound	• Localized damage • Clinically relevant • Technically simple	• Difficult to precisely reproduce • Rarely encountered clinically	Brain	[115]
				SC	N/A
	Transection	• Localized lesion • Complete axon cut • Consistent and reproducible • Clinically relevant	• Open dura • Rarely encountered in clinically • Gap between tissues	Brain	N/A
				SC	[116, 117]
	Hemisection (Dorsalor Lateral)	• Clinically relevant • Targeted interruption of spinal tracts • Potential for within-subjectcontrol (e.g., intact side of lateral hemisection) • Selective tract targeting	• Rarely encountered clinically • Difficult to precisely reproduce injury location/depth	Brain	N/A
				SC	[118]
	Compression	• Similarto crush injury • High clinicaly relevance • Possibility to control damage severity	• Difficult to precisely reproduce consistent severity	Brain	N/A
				SC	[56, 119, 120]
	Contusion	• Most clinically relevant • Less invasive • Consistent central lesion and inflammation	• Requires specialized equipment (impactor) • More variability than other models	Brain	[121]
				SC	[122]
	Hypoxic-ischemic Injury or Stroke	• High clinical relevance	• Induces secondary damage from ROS, and excitotoxicity • Not localized	Brain	[123–132]
				SC	N/A
	Cryogenic	• Control over lesion extent • Requires only simple instruments	• Variable	Brain	[57]
				SC	N/A
	Irradiation	• Allows to target specific regions • Dose can be adjusted to control injury severity	• Limited accessibility to specialized lasers • Potential secondary radiation injury	Brain	[133–135]
				SC	N/A

## Homeostatic and regenerative neurogenesis in zebrafish and neonatal mice

### Telencephalon

The telencephalon constitutes the most significant portion of the brain and, like in humans, consists of two distinct hemispheres in both zebrafish and mice. This brain region controls motor and sensory information, conscious and unconscious behaviors, feelings, intelligence, and memory [136].

**Fig. 2:**
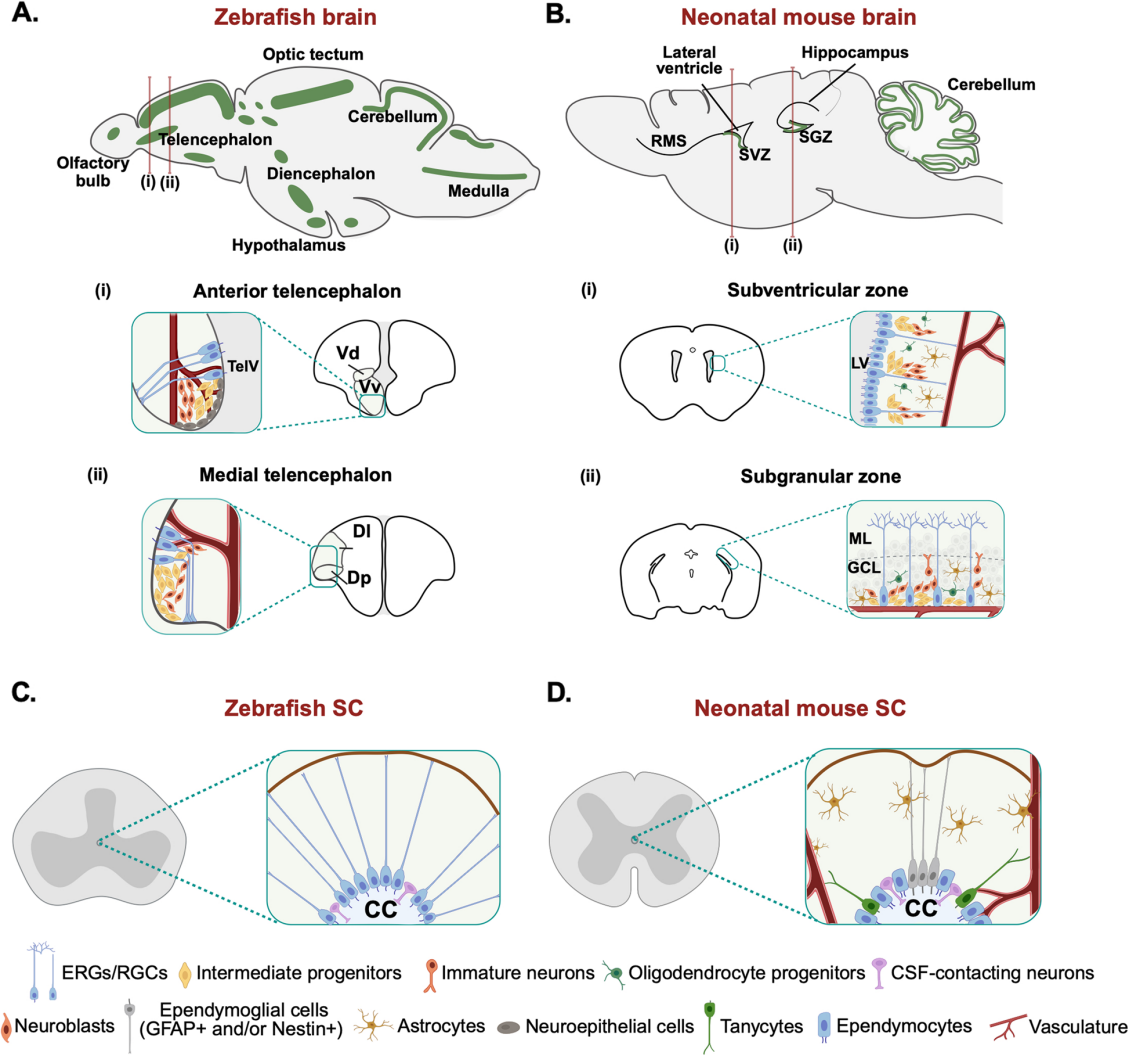
Neurogenic niches in the central nervous system of zebrafish and neonatal mice. **A** Sagittal section of the zebrafish brain showing the 16 neurogenic niches in green (top panel). Coronal sections are taken through line (*i*) and (*ii*). (*i*) highlights the Vd-Vv region, equivalent to SVZ of mammals; (*ii*) highlights the Dl-Dp region, equivalent to the mammalian hippocampus. The insets show the cellular arrangement in each region. RGCs extend long projections toward the pial surface. When activated, they proliferate to give rise to intermediate progenitors and neuroblasts. These migrate to their prospective locations and differentiate into neurons. **B** Sagittal section of the neonatal mouse brain showing neurogenic niches in green. Lines represent coronal sections through (*i*) and (*ii*). (*i*) highlights SVZ adjacent to the lateral ventricles, with RGCs in contact with blood vessels. RGCs proliferate to form intermediate progenitors and neuroblasts that migrate to the olfactory bulb through the RMS, to then differentiate into olfactory neurons. (*ii*) SGZ in the dentate gyrus of the hippocampus, whose cell layers can be broadly divided into the molecular layer (ML) and the granular cell layer (GCL). Local RGCs produce hippocampal granule neurons that integrate into the hippocampal circuitry through intermediates. **C** Transverse section of the zebrafish spinal cord. Inset shows the arrangement of ERGs and cerebrospinal fluid (CSF)-contacting neurons around the central canal. **D** Neonatal mouse spinal cord cross-section with inset showing ependymocytes, tanycytes, ependymoglial cells (GFAP + and/or Nestin +), CSF-contacting neurons, and free astrocytes surrounding the central canal. TelV, Telencephalic ventricle; Vd, Dorsal nucleus of ventral telencephalonic area; Vv, Ventral nucleus of ventral telencephalonic area; Dl, Dorsal telencephalon lateral zone; Dp, Dorsal telencephalon posterior zone; RMS, Rostral migratory stream; LV, Lateral ventricle; ML, Molecular layer; GCL, Granular cell layer; CC, Central canal. Figure generated using Biorender

Although the zebrafish brain contains sixteen neurogenic niches (Fig. 2A), a substantial body of research has focused on the dorsal telencephalon. This region hosts the territories homologous to the mouse ventricular-subventricular zone (V-SVZ), located in the walls of the lateral ventricles, and the sub-granular zone (SGZ) of the dentate gyrus in the hippocampus. Together with the hypothalamus [137, 138], the V-SVZ [139, 140] and SGZ [141, 142] are the main regions of active neurogenesis in the adult rodent brain (Fig. 2B). Non-invasive in vivo imaging and single-cell lineage tracing studies in zebrafish show that telencephalic neural progenitor niches are mostly composed of radial glial cells (RGCs) and non-glial cycling neuroblasts, both lining the ventricle. The RGCs are neural stem cells able to generate new RGCs and neurons through a series of intermediate, amplifying, and non-glial cell states. Quiescent (type 1) and proliferative (type 2) RGCs form a closely packed single layer, with their cell bodies aligned along the ventricle [143]. They display apico-basal polarity, with a region facing the cerebrospinal fluid and a lengthy, extensively branched basolateral projection extending throughout the parenchyma, and typically express markers associated with astroglia, such as glial fibrillary acidic protein (GFAP), brain lipid-binding protein (BLBP), nestin, glutamine synthetase (GS), and S100β [143–145]. They also express progenitor markers, such as the transcription factors SRY-Box 2 (SOX2), HES related family BHLH transcription factor with YRPW motif 1 (HEY1), and Hairy-related 4 (HER4, mouse HES5) [53, 146, 147]. Non-glial cycling neuroblasts (type 3) can be found tightly inserted between RGC soma and are proposed to originate from RGCs. They undergo a limited amplification phase before performing symmetric neurogenic divisions and are similar to the mouse transit-amplifying progenitors [144]. Neuroblasts are subdivided into type 3a and type 3b. Both types express proliferating cell nuclear antigen (PCNA) and polysialylated neuronal cell adhesion molecule (PSA-NCAM), while type 3a cells also express some RGC markers [144].

The zebrafish telencephalon undergoes basal, constitutive neurogenesis during homeostasis originating from RGCs and non-glial cycling neuroblasts [53, 68] (Fig. 2A). Following mechanical injury, a larger pool of RGC progenitors is activated [148, 149]. Perilesional RGCs proliferate, peaking at 7 days post-injury, before gradually returning to their baseline proliferation levels. Genetic lineage tracing combined with bromodeoxyuridine (BrdU)-mediated labeling of cycling RGCs showed that they generate neurons able to migrate toward the lesion site and integrate into the pre-existent functional circuit while establishing synaptic connections. A key distinction between neurons generated during homeostasis and those produced in response to injury is that the latter exhibit enhanced migratory abilities, enabling them to contribute to the repair of damaged tissue [68, 150].

Neurogenesis in the neonatal mouse telencephalon is relatively understudied. Two studies found that following either brain cryoinjury or ischemic stroke, the neonatal rodent V-SVZ produces a larger number of neuroblasts compared to adults [123, 151]. In agreement with this, Foucault et al. recently used chronic hypoxia to induce brain damage and demonstrated reactivation of glutamatergic progenitors, which parallels cortical neurogenesis [124]. Neonatal neuroblasts possess a higher migratory capacity than the adult counterpart, which has been at least partially attributed to RGC fibers. While these fibers generally disappear soon after birth, they persist in injured neonatal mouse brains and act as a scaffold for postnatal neuroblasts to migrate toward the lesion site, enabling cellular and functional regeneration of neurons. This process depends on N-cadherins, which form cell adhesion structures between RGCs guiding neonatal neuroblast migration via saltatory movements [57]. In mammals, bona fide RGCs do not persist into adulthood. However, constitutive neurogenesis originating from radial glia-like cells has been observed in the adult mouse V-SVZ. Here, proliferating radial glia-like cells give rise to transient amplifying cells, which in turn generate neuroblasts that continuously migrate through the rostral migratory stream to the olfactory bulb, where they differentiate mature neurons that process olfactory input [139, 152, 153]. In the adult SGZ, proliferating radial and non-radial precursors give rise to intermediate progenitors, which in turn generate neuroblasts (Fig. 2). Immature neurons migrate into the inner granule cell layer of the dentate gyrus and differentiate into neurons that process information relevant to learning and memory in the hippocampus [141, 142, 154]. In a recent study, Kokeva et al. demonstrated that new cells are born continuously and in substantial numbers in the adult murine hypothalamus and that many of these cells differentiate into neurons as indicated by the expression of doublecortin and other neuronal markers [155]. Several studies have shown that TBI in the mature rodent brain activates endogenous neural stem cells in the neurogenic regions described above, as expertly reviewed elsewhere [156–159]. However, the overall pool of newly generated neurons after injury in adult mice is smaller compared to both neonatal mice and zebrafish.

### Cerebellum

The cerebellum, located in the hindbrain, possesses a highly intricate folded structure that accommodates the largest concentration of neurons in the brain. It plays a crucial role in maintaining balance, orchestrating precise motor coordination and facilitating advanced cognitive functions. The cerebellum in zebrafish and mammals exhibits a high degree of similarity, with conserved neuronal cell types, circuitry, physiology, and functionality [160, 161].

Two neurogenic niches have been identified in the zebrafish cerebellum, each giving rise to different neuronal subtypes during homeostasis. The first niche, located in the ventricular zone (VZ), contains cells with RGC-like morphology responsible for generating Purkinje cells, mossy fiber neurons, climbing fiber neurons, and interneurons. The second niche is a region within the upper rhombic lip, where neuroepithelial-like cells give rise to nestin-expressing granule cells. While homeostatic neurogenesis ceases following the juvenile stage in the VZ niche, it persists throughout life in the rhombic lip [162, 163]. Injury in adult zebrafish induces activation and proliferation of both neuroepithelial-like cells and RGC-like progenitor niches. However, the number of injury-activated RGC-like progenitors remains low, even at the peak of activation, indicating that they play a minor role in adult cerebellar neurogenesis after injury compared to neuroepithelial-like cells [162, 163].

Reports on the regeneration of Purkinje cells in the adult zebrafish cerebellum are conflicting [162, 164, 165]. Studies employing mechanical ablation of cerebellar regions suggested that zebrafish Purkinje cells only regenerate at larval stages, with this capacity lost by 3 months of age due to depletion of the progenitor pool [163]. In contrast, a study employing the ATTAC model to selectively ablate Purkinje cells via tamoxifen administration shows that Purkinje cells regenerate in both larval and adult zebrafish. Importantly, the regenerated Purkinje cells are functional and enable the reacquisition of cerebellum-controlled behavior [106]. Although permanent and inducible lineage tracing of possible Purkinje cell progenitors has not yet been performed, co-localization studies suggest that new Purkinje cells do not originate from radial glia-like cells. Instead, neuroepithelial-like cells expressing pancreas-associated transcription factor 1a gene (*ptf1a*) might play a major role in this process [106]. These findings highlight the heterogeneity of stem cell proliferation patterns and fate commitment in the zebrafish cerebellum.

Similarly, both granule and Purkinje cells regenerate in the cerebellum of neonatal mice. Cerebellar granule cells are generated by intensive cell division of granule cell precursors during postnatal development. Depletion of the granule cell precursor pool by radiation exposure or genetic ablation leads to generation of new granule cells via the adaptive reprogramming of gliogenic nestin-expressing progenitors located in the Bergmann glia layer [107]. Upon damage, these nestin-expressing progenitors undergo YES-associated protein (YAP)-dependent migration toward the injury site and transiently upregulate expression of *Ascl1* and *Atoh1*, which encode achaete-scute family BHLH transcription factor 1 and atonal BHLH transcription factor 1 respectively. Both transcription factors orchestrate adaptive reprograming of nestin-expressing progenitors into granule cells [133, 134].

Regeneration of Purkinje cells also occurs following early postnatal loss in mice. In an insightful study, Bayin et al. employed a combination of diphtheria toxin-mediated Purkinje cell ablation and fate-mapping strategies to identify a population of immature Purkinje cells in the injured cerebellum that can divide to replace lost Purkinje cells. These immature Purkinje cells are marked by forkhead box protein 2 (FOXP2), normally expressed by embryonic Purkinje cells, and lack expression of the Purkinje cell marker calbindin 1 (CALB1). Interestingly, this regenerative process occurs only when Purkinje cells are ablated at postnatal day 1. The mechanisms driving pro-regenerative responses of immature Purkinje cells require further investigation [135].

### Spinal cord

In the zebrafish spinal cord, most neurons are generated between 14- and 48-hours post-fertilization [166, 167]. Little neurogenesis occurs after this period, with almost no neurogenic events observed during homeostasis in adults [84]. Injury at either larval or adult stages triggers spinal neurogenesis by activating ependymo-radial glial cells (ERGs), which form the main spinal cord neuronal progenitor pool [64, 84, 168]. Also known as “tanycytes,” “ependymo-glia,” and “radial glia,” ERGs possess a dual identity, exhibiting features of both ependymal cells surrounding the central canal and RGCs described in the brain [84, 169, 170]. They comprise one or two cilia [65] and are characterized by expression of the transcription factor FOXJ1a [66]. Upon injury, spinal cord ERGs display properties and functions similar to those described for telencephalic RGCs [169]. Injury-induced activation of ERGs begins with a proliferation phase that depends on the epigenetic modulator Sirtuin 1 (SIRT 1) [85]. Then, depending on their position around the central canal and expression profile, ERGs give rise to specific neuronal cell types, such as serotonergic neurons, interneurons, and motor neurons. This process is highly controlled and results from an intricate balance of pro-neurogenic and anti-neurogenic signals. These range from growth factors (Heparin binding growth factor a (HB-EGFa), Myostatin) [86, 171], to developmental signals (NOTCH, Hedgehog (HH), Bone morphogenetic protein (BMP), Wingless-related integration site (WNT)) [169, 172], to neurotransmitters [87, 88, 173, 174], all able to influence activity of resident spinal progenitor cells. In a 2021 study, Vasudevan et al. used genetic, electrophysiological, and behavioral approaches to examine the identity and physiology of interneurons generated after SCI [74]. The authors showed that newly generated cells expressing a transgenic marker of premotor V2a interneurons receive synaptic inputs and fire synchronously with evoked motor output at 9 days post-injury. These findings suggest that ERGs display a high level of spatial complexity in the zebrafish spinal cord and give rise to neuronal subpopulations after injury that integrate in existing circuits to restore locomotion [74] (Fig. 2C). Vandestadt et al. recently showed that in zebrafish larvae, embryonically derived precursor neurons are recruited to the lesion site before ERG-derived neurogenesis begins. These cells appear to form the initial functional circuitry underlying spinal cord repair [75]. A population of injury-surviving neurons that acquire a neuroblast-like gene expression signature after injury has also been recently reported in adult zebrafish [89]. Regardless of their origin, newly generated neurons successfully integrate into the locomotor circuitry to refine functional recovery, receive excitatory input, and fire synchronously with motor output [74]. Notably, the successful regeneration of the zebrafish spinal cord has been mostly attributed to its high neurogenic capacity. However, an elegant recent study by Pedroni et al. showed that particularly vulnerable neurons in the mammalian spinal cord, such as motoneurons, remain resistant to damage in zebrafish [90]. This finding introduces the concept of neuroprotection to the already complex story of spinal cord neuroregeneration, raising new questions about mechanisms enhancing it in zebrafish.

Unlike in zebrafish, neurogenesis in the regenerating mouse spinal cord has not been reported. Yet, the adult mouse spinal cord is proposed to harbor endogenous stem cells around the central canal [175–177]. This region is populated by different types of highly polarized ependymal cells, which can be distinguished both morphologically and by their marker expression. The main cell type found around the central canal are ependymocytes, ciliated cells with a cuboidal morphology. Tanycytes (also referred to as radial ependymocytes) are also frequently observed, comprising long processes that extend to the basal lamina of blood vessels [178, 179]. The dorsal and ventral regions of the central canal display cells with a long radial morphology expressing GFAP and/or Nestin. Because of their radial morphology, these cells are often considered a subtype of tanycytes [180, 181]. In addition to ependymal cells, cerebrospinal fluid-contacting neurons are also sporadically distributed around the central canal (Fig. 2D). The presence of cells with radial morphology around the central canal of mice suggests that the ependymal zones in zebrafish and mammals may share more similarities than previously thought. Remarkably, in both zebrafish and mice, cells in the ependymal region express the stemness factor SOX2. However, while ERGs in the zebrafish ependymal layer give rise to neurons, this potential is restricted in mice. In uninjured neonatal mice, proliferation occurs in cells around the central canal and mostly involves bi-ciliated ependymocytes. This process declines when the spinal cord reaches its final size at 12 weeks of age [182]. To date, only one study by Chawla et al. investigated whether reparative neurogenesis occurs in neonatal mice. The authors performed thoracic compression injury in neonatal mice at postnatal day 1, finding an increase in cell proliferation with no significant differences in neurogenesis. Recovery of motor function after injury in neonatal mice was attributed to recovery of both excitatory and inhibitory terminals, as well as of serotonergic innervation below the injury site, rather than neurogenesis itself [56, 119, 182].

Several studies have investigated the stem cell potential of cells around the central canal in adult mice after SCI. Injury increases the expression of GFAP, cell fate regulators, and progenitor cell markers such as NOTCH 1, NUMB, PAX6, SHH, BMP and MSX2 [183–186] in cells in the ependymal region. In a recent study, Albors et al. characterized the transcriptome of these cells at the single cell level, finding that with age, cells located laterally around the central canal shift toward a more mature state. However, immature cells remain in significant proportions in the aged ependymal region. The authors propose that these cells may function as stem cells following SCI [187]. The stemness potential of ependymal cells was first demonstrated in 1996 by Weiss et al., who showed that spinal neurospheres derived from adult mice could differentiate into astrocytes, neurons, and oligodendrocytes [188]. The number of neurospheres obtained using injured spinal cords was higher than those of uninjured mice [186, 189, 190]. After injury, these neurospheres mostly form oligodendrocytes, and the amount of both neurospheres and oligodendrocytes is higher in cultures of juvenile versus adult ependymal cells [190]. In vivo, after SCI in adult mice, bi-ciliated ependymocytes proliferate, migrate to the lesion site, and generate scar-forming astrocytes, as well as a smaller number of oligodendrocytes [189, 191, 192]. Therefore, while spinal cord-derived neurospheres can generate neurons in vitro, progenitor cells predominantly give rise to glial cells in vivo. Supporting the potential of ependymal cells to generate oligodendrocytes, a 2022 study by Llorens-Bobadilla et al. discovered that chromatin regions containing binding motifs for the oligodendrocyte-determining transcription factors OLIG2 (oligodendrocyte transcription factor 2) and SOX10(SRY-Box Transcription Factor 10) became accessible in ependymal cells after SCI, even though these transcription factors were not expressed. When OLIG2 was overexpressed in ependymal cells in vivo, it significantly enhanced the accessibility of OLIG2 binding sites and promoted the production of oligodendrocytes from ependymal cells following SCI [193]. These findings raise the possibility that endogenous ependymal cells in the mouse spinal cord could be engineered or manipulated to produce neurons after SCI. However, stem cell potential of ependymal cells remains controversial. Some studies suggest that cells in the mouse ependymal region are not a major source of endogenous neural stem cells or neuroprotective astrocytes after SCI, given their restricted differentiation potential and limited migration capacity [194, 195]. These contrasting findings provide evidence that large spinal cord lesions contain many newly proliferating cells, but few of them are positive for the ependymal lineage marker FOXJ1. Ependymal progeny is generated in small numbers only after direct damage, and these reside locally in the immediate peri-ependymal area [194]. Thus, further analyses are needed to clarify whether the cells in the mouse ependymal layer possess true stem cell potential and how they compare to zebrafish ERG progenitors.

### Mechanisms of axon regeneration in zebrafish and neonatal mice

Zebrafish possess a remarkable capacity to regenerate axons. Following spinal cord transection injury, Becker and colleagues were the first to observe axon regrowth from the brainstem that transverses the lesion site and promotes functional motor recovery in adult zebrafish [52, 91]. This regenerative capacity is also evident at larval stages, as extensively reviewed by Tsata and Wehner [196]. Although still a matter of debate, glial bridges have been proposed to provide a substrate for regenerating axons to cross the lesion site. Indeed, specialized glial cells expressing connective tissue growth factor a (CTGFa) after injury have been shown to undergo a YAP-dependent epithelial to mesenchymal transition after SCI in adult zebrafish. These cells then localize at injury sites, bridging the two severed stumps and supporting regrowing axons [92, 93]. In line with this model, genetic ablation of CTGFa + bridging glia in adult zebrafish led to a reduction in axon regeneration and functional recovery [94]. The requirement of a glial bridge for axon regeneration in zebrafish is still a controversy in the field. Studies in larvae employing a genetic system to ablate GFAP + glia have demonstrated that axonal regeneration precedes migration of glia at the lesion site and can occur independently of glial cell involvement [76]. Although these data suggest that mechanisms of axon regeneration might differ with aging, further clarification is needed to determine whether bridging glia may be required in adult zebrafish but not in larvae. Another consideration is that CTGFa in the adult injured spinal cord is not specific to bridging glia only. In fact, it is also expressed by other cell types surrounding injury sites as well as in the cord ventral domain, which harbors sonic hedgehog (SHH) neuronal progenitors [92–94, 169]. Disrupting *ctgfa* gene expression in these cells and/or domains might per se affect axon regeneration. On the other hand, the genetic ablation systems that deplete GFAP + glia in larvae may lead to incomplete and/or non-specific cell ablation, creating a scenario where a minor fraction of GFAP + glial cells might have escaped ablation in the larval experiments and could still support axonal regrowth [94]. In zebrafish, the number of regenerated brain-derived long descending axons and intraspinal long-projecting interneuron axons following SCI is only one-third of that observed in uninjured animals. Over 80% of regrown axon-bearing neurons are glutamatergic excitatory interneurons, while glycinergic inhibitory interneurons account for less than 10%. The excitatory-to-inhibitory ratio shifts from 2:1 to 10:1 after injury, reflecting a reorganization of spinal central pattern generator circuits. The regrowth of interneurons is dependent on a specialized population of intraspinal serotonergic neurons appearing after injury. These cells form varicosities that continuously release serotonin, activating 5-HT1B receptors to re-establish the central pattern generator, which is responsible for generating rhythmic movements needed for locomotion [95]. Most of the regrown excitatory neurons are V2a interneurons. They form a modular circuit where fast and slow V2a interneurons rostral to the lesion selectively synapse fast and slow V2a/motor neurons caudal to it [96].

Axon regeneration and re-establishment of synaptic connectivity have also been described in neonatal mice after SCI and shown to decline with age [118]. Employing a newly developed thoracic compression injury model, Boulland et al. observed that neonatal SCI was accompanied by hindlimb paralysis and that axons and synaptic connections began to regenerate as early as 24 h post-injury. Unlike in adult mice, this occurred without astrogliosis. By 7 days post-injury, restoration of hindlimb movement began, descending input reappeared below the injury site, and both excitatory and inhibitory synaptic terminals were recovered [56, 119]. This recovery was also observed after lumbar injuries [120]. Both intrinsic and extrinsic mechanisms are thought to contribute to the decline in axon regenerative capacity as neurons mature and age [197]. In a 2010 study, Liu et al. showed that immature mouse neurons with high regenerative ability have high intrinsic levels of mammalian target of rapamycin (mTOR) activity and that inactivation of the upstream phosphatase and tensin homolog (PTEN) promotes regeneration of corticospinal tract axons that can reform synapses in the spinal cord caudal to the lesion site [195, 198]. Subsequently, although functionally redundant in development, inhibition of zebrafish PTENa, but not PTENb, enhances regeneration in adult zebrafish with SCI [199]. These findings highlight consistencies in intrinsic factor conservation between fish and mammals. Interestingly, mTOR activity undergoes a development-dependent downregulation in many types of CNS neurons, including corticospinal neurons, suggesting that lack of mTOR activation is a general intrinsic mechanism underlying the diminished regenerative ability in the adult mouse CNS. Manipulation of intrinsic neuron growth programs via deletion of growth inhibitory PTEN and suppressor of cytokine signaling 3 (SOCS3) or overexpression of growth promoting factors like insulin-like growth factor 1 (IGF1) and osteopontin have been proposed as essential elements of combinatorial approaches to enhance the limited regenerative potential of adult CNS neurons [200].

Additional insight into the intrinsic signals contributing to the loss of regenerative capacity with aging come from studies on mammalian retinal ganglion cells. For instance, Wang et al. recently demonstrated that overexpression of the histone methyltransferase enhancer of zeste homolog 2 (*Ezh2*) in CNS retinal ganglion cells promotes optic nerve regeneration through both histone methylation–dependent and –independent mechanisms. Gene expression profiling revealed that EZH2 promotes regeneration by suppressing expression of maturation-associated genes, including those encoding ion channels, transporters, and neurotransmitter receptors and genes inhibiting axon regeneration [201]. Changes in methylation patterns with aging have also been attributed to differences in regenerative ability between neonatal and adult mice. An elegant 2020 study by Hoffman et al. showed that overexpression of the “Yamanaka transcription factors” OCT4, SOX2, and KLF4 (collectively known as OSK) in adult mouse retinal ganglion cells restores youthful methylation patterns and promotes axon regeneration. This process is dependent on Tet methylcytosine dioxygenase 1 and 2 (TET1 TET2) activity, whose knockdown abrogates the ability of OSK overexpression to stimulate axon regeneration [202]. These findings indicate that mammalian tissues retain a record of youthful epigenetic information, encoded in part by DNA methylation. In a recent preprint, Ruven et al. investigated the precise developmental time at which the CNS loses its capacity for long-distance axon growth [116]. The authors developed a surgical method to meticulously axotomize corticospinal tract axons in the neonatal spinal cord, without damaging their microenvironment. When corticospinal axons in mice were injured on postnatal day 1, during elongation, they regenerated. Specifically, axotomy at thoracic T2 level resulted in 16.1 ± 2.4% of axons reaching T5, and 12.6 ± 2.7% reaching L2, a rate statistically indistinguishable from non-lesioned controls. However, when axons were injured on postnatal day 4, after they had arborized in the spinal cord, they failed to regrow. The transition from elongation to arborization occurs gradually, with axons in rostral spinal cord segments undergoing this transition earlier than those in caudal segments. Notably, corticospinal axons injured at more caudal levels (e.g., thoracic) on postnatal day 4 could still regenerate, indicating that regenerative capacity is lost as development progresses. In this study, long distance corticospinal axon growth did not correlate with astrocytic and microglial activation, nor with myelination levels. This has been attributed to the minimal tissue disruption caused by the microlesions, compared to more substantial crush injury models in neonatal mice [116].

External to the neurons, recent research suggests that regenerating axons may use a scaffold to navigate the lesion site, as observed in adult zebrafish. In mice, this scaffold is made of fibronectin, secreted by microglia accumulating at the lesion site. Depletion of microglia in mice using PLX 3397, a colony-stimulating factor 1 receptor (CSF1R) inhibitor, impaired fibronectin bridge formation between the two stumps at 3 or 7 days post-injury, with most axons stalled at the lesion epicenter. The same was observed after postnatal day 2 injury in mice with conditional knockout of CSF1R, which removed about 70% of microglia [113], and in transgenic mice allowing microglia-specific deletion of the fibronectin gene. The accumulation of fibronectin and other extracellular matrix (ECM) components (i.e., Collagen XII) at the lesion site in close association with regrowing axons has also been documented by Wehner et al. in the larval zebrafish spinal cord [76]. Hence, as described in the following sections, axon-ECM interactions might be important for axonal re-growth in both zebrafish and neonatal mice. The involvement of extrinsic mechanisms in the decline of axon regenerative capacity observed with aging has also been documented in another study from Geoffroy et al., using mice carrying a deletion in the PTEN, a manipulation known to promote regeneration of retinal ganglion and corticospinal tract axons after injury. The authors showed that the regeneration-promoting effect of PTEN deletion is greatly diminished with aging in both the corticospinal and rubrospinal tracts. PTEN deletion in older animals remains effective in elevating neuron-intrinsic growth states, as assessed by mTOR activity, neuronal soma size, and axonal growth proximal to the injury site [118]. This suggests that an increased level of negative environmental influence at the injury site in aging mice is at least one underlying mechanism for regeneration failure. Further supporting this evidence, a recent preprint has attributed differences in regenerative capacity between neonatal and adult mice to anatomical factors, particularly the proximity of the injury to the neuron’s cell body, rather than intrinsic cellular characteristics [203]. The authors found that SCI leads to minor transcriptional changes in mixed neuronal supraspinal populations and corticospinal tract neurons, as evidenced by the number of affected transcripts and their degree of up- or down-regulation. Conversely, axotomy near corticospinal tract neuron cell bodies resulted in significantly greater transcriptional effects, indicating that the location of injury relative to the neuron’s cell body plays a crucial role in determining the extent of the transcriptional response and regeneration. This anatomical difference may help explain the enhanced regenerative capacity observed in neonatal mice and zebrafish, where the distance between the cell body and axonal injury is smaller than in adult mice [203]. Whether axon regeneration in neonatal mice involves a bridging glia population, as seen in zebrafish, and how these processes overlap between species remains an open question.

Additionally, the loss of CNS axon regenerative capacity coincides with neuronal maturation and the initial formation of synapses. Proteins involved in synapse formation, synaptic transmission, and myelination have all been proposed as further extrinsic factors involved in the loss of regenerative capacity with aging. For instance, when axons are injured or deprived of activity, they are prevented from regenerating by oligodendrocyte precursor cells that entrap them in synapse-like structures [204], and blocking myelination restores some regenerative capacity in the visual system. Understanding the relationship between the synapse and axon growth in mature neurons remains a critical area for future research. Synaptic vesicle release dynamics might also suppress axonal regrowth, as neurons mature during development. In agreement with that, deletion or silencing of *Cacna2d2*, the gene coding for the alpha 2 delta 2 subunit of voltage-gated calcium channels regulating synaptic neurotransmitter release, promotes axon regeneration [205]. More recent evidence shows that genetic loss-of-function of *Munc13s*, coding for a key component of the synaptic transmission machinery, also promotes axon regeneration after CNS injury in adult mice [206]. Neurotransmitter pools released after injury may also contribute to the differences in regenerative capacity between neonates and adults. Indeed, SCI in adults shifts excitatory interneurons to an inhibitory phenotype, reducing synaptic excitation to motor neurons. In contrast, in neonatal injury, excitatory interneurons preserve an excitatory phenotype, which is essential to restore motor function. Mimicking the adult inhibitory phenotype in neonates impairs locomotor function [117]. Neurotransmitter phenotype switch also occurs during zebrafish regeneration, suggesting that this may be a key mechanism underlying axon regeneration [207]. These findings suggest that the regenerative potential of neonatal neurons is associated with their immature state and limited synaptic activity.

## Common signals promoting CNS regeneration in zebrafish and neonatal mice

**Fig. 3:**
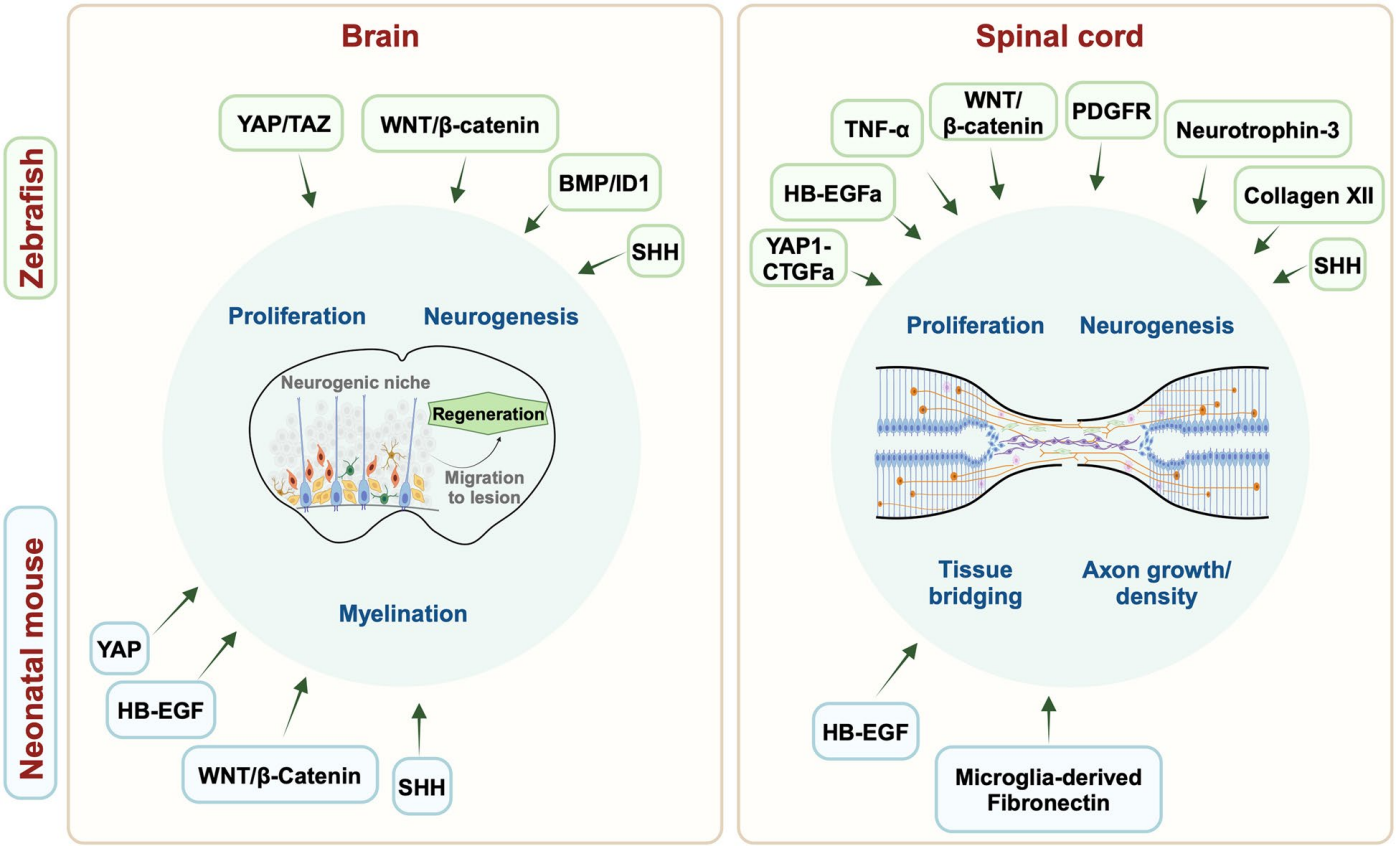
Pathways and cell types regulating CNS regeneration in both zebrafish and neonatal mice. The circles show a schematic of the major processes involved in the regeneration of the zebrafish and neonatal mouse brain and spinal cord. Highlighted are signaling pathways, molecules and cell types that play, or are likely to play, a role in regeneration in both zebrafish (green rectangle) and neonatal mice (light blue rectangle). Figure generated using Biorender

Over the past decade, a growing number of studies have employed zebrafish and neonatal mice to identify signals promoting innate brain and spinal cord repair. Below, we provide an overview of factors and signals that may be crucial for regeneration in both organisms (Table 2, Fig. 3).

**Table 2:** Common signals relevant to brain and spinal cord regeneration in both zebrafish and neonatal mice

Pathway	Model	Induction domain and/or cell type	Evidence for regeneration	Ref
**HIPPO**	Zebrafish	Brain progenitor cells, spinal cord ERGs	• Increased neural progenitor proliferation and neuronal differentiation • Epithelial to mesenchymal transition of ERGs into bridging glia	[93, 208]
	Neonatal Mice	Cerebellar neuroepithelial-like cells	• Increased granule cell migration • Improved cerebellar growth	[134]
**SHH**	Zebrafish	Cerebellar progenitors, spinal cord ERGs	• Enhanced proliferation of neural progenitors • Increased motoneuron regeneration in SC • Improved motor recovery	[67, 169, 209]
	Neonatal Mice	Progenitors of V-SVZ and cerebellum	N/A	[210–213]
**WNT**	Zebrafish	Brain ependymoglia and spinal cord ERGs	• Increased neural progenitor proliferation and differentiation into neurons • Improved axonal bridging in SC	[67, 69, 70, 88, 97, 98, 214]
	Neonatal Mice	Brain NSCs	• Increased NSC proliferation and neuronal differentiation • Reduction of NSC apoptosis	[124, 125, 215, 216]
**BMP/Id1**	Zebrafish	BMP: Neurons Id1: NSCs	• Prevents NSC pool depletion • Maintains long-term regenerative capacity	[71]
	Neonatal Mice	Multipotent neural progenitor cells of brain	• Downregulation improves OPC survival and white matter protection	[126, 127, 217–219]
**HB-EGF**	Zebrafish	ERGs	• Neurogenesis and axon growth	[86, 220, 221]
	Neonatal Mice	N/A	• Myelin preservation in brain • Increased 5-HT axons density in SC	[86, 128]
**ECM factors**	Zebrafish	Fibroblast-like cells of myoseptal and perivascular origin	• Collagen XII: Increased axonal guidance and bridging in SC • Pdgfrb: Increased axon bridging, reduced scarring in SC	[76, 77, 99]
	Neonatal Mice	Microglia	• Fibronectin: Forms a bridge across the lesion site supporting regenerating axons	[113, 129]
**Immune cells**	Zebrafish	zT_reg_ cells, blood-derived macrophages, microglia	• Cause increased ERG proliferation and axonal sprouting in SC through Neurotrophin-3 and other neurogenic factors • Cause reduction of neutrophil-derived proinflammatory cytokines • Promote neurogenesis and axon regeneration by modulating TNF-α levels • Effectively clear debris	[72, 73, 78–81, 100, 108, 222]
	Neonatal Mice	Blood-derived macrophages, microglia	• Clearance of myelin and cellular debris • Fibronectin bridge formation in spinal cord • Injury alleviation, reduction of scarring	[113, 114, 130, 131, 223, 224]

### Hippo signaling

Hippo signaling plays a crucial role in both development and regeneration. In the zebrafish brain, effectors of the Hippo signaling pathway—YAP, TAZ (transcriptional co-activator with PDZ-binding motif), and TEAD (transcriptional enhanced associate domain)—control progenitor cell proliferation. They are expressed in mechanosensory receptors that detect actomyosin tension along the developing hindbrain. Reduced YAP/TAZ-TEAD activity leads to decreased neuronal differentiation [208]. Hippo signaling also plays a role during zebrafish spinal cord regeneration. Specifically, for a glial bridge to form, ERGs must transition to a mesenchymal cell phenotype and migrate to the injury site. This process is regulated by YAP1-CTGFa signaling, as both these genes are upregulated following injury and localize near the lesion. Moreover, YAP1 is known to increase expression of *twist1a*, a marker of epithelial to mesenchymal transition [93]. Loss of function of *twist1a* impairs bridging and motor recovery, while its overexpression or administration of human recombinant CTGF to spinal lesions restores baseline functional outcomes [93].

Some studies suggest that Hippo signaling might also play a role in the neonatal mouse CNS, specifically during regeneration of the cerebellum. Neuroepithelial-like cells (major stem cell pool within the cerebellum that gives rise to granule cell precursors) [133] are enriched with YAP and TAZ in neonatal mice. Yang et al. showed that mutation of YAP specifically in neuroepithelial-like cells at postnatal day 0 results in reduced granule cell migration at postnatal day 12 and cerebellar growth at postnatal day 16, following cerebellar irradiation at postnatal day 1. TAZ mutation, however, did not corroborate these findings. Rather, when both YAP and TAZ were mutated in neuroepithelial-like cells, the cerebellar phenotype was not greatly affected [134]. This suggests that loss of TAZ may rescue deficits caused by YAP mutation, highlighting the complex and potentially compensatory roles of these proteins in cerebellar regeneration.

While Hippo signaling has not been studied in neonatal spinal cord regeneration, studies in adult mice revealed that YAP is upregulated following injury and modulates astrocytic proliferation, aiding in the formation of a glial scar. It is also worth noting that inhibiting YAP or expression of its target gene *Ccna2* prevented scar formation [225, 226]. While study of Hippo signaling in pro-regenerative, neonatal mice still awaits, these findings suggest that the consequences of Hippo activation are possibly genetically, temporally, and species dependent.

### Sonic Hedgehog signaling

Developmentally, Shh signaling is thought to control cell differentiation patterns, and its dysregulation is associated with neurodegenerative diseases [227].

In zebrafish, blunt force trauma to the cerebellum was shown to induce a significant upregulation of the SHH ligands (SHHa and SHHb) at 6 hours post-injury and of its receptor Smoothened at 12 hours post-injury, both of which returned to baseline levels by 60 hours post-injury [209]. In the same study, activation of this pathway via administration of the Smoothened agonist purmorphamine in uninjured fish triggered a proliferative response along the cerebellar crest which ultimately contributed to populating the granule cell layer of the cerebellum. Further, injection with the Shh antagonist cyclopamine following TBI resulted in the absence of a proliferative response [209]. In the adult zebrafish spinal cord, SHH is constitutively expressed at a basal level in ERGs but significantly upregulated following injury, particularly in ventrally-located ventrally ERGs around the central canal, with expression peaking around 3 days post-injury [67]. When SHH signaling was inhibited via intraperitoneal injections of cyclopamine, ERG proliferation decreased, which correlated with impaired regeneration of motor neurons [169].

In neonatal mice, SHH exerts both proliferative and pro-neurogenic effects. Specifically, during the first week of life, SHH signaling is required to maintain high oligodendrocyte proliferative capacity in the dorsal V-SVZ of the brain [210]. Although this innate capacity is lost in adults, similar rates of oligodendrocyte proliferation are observed when the pathway is ectopically reactivated in mice at postnatal day 30. In the neonatal mouse cerebellum, genetic or pharmacological disruption of SHH signaling alters the angles of spindle orientation, which in turn affects the balance between symmetric and asymmetric granule cell progenitor divisions [211]. The contribution of SHH signaling to neonatal mouse spinal cord regeneration has not been studied, but therapeutic interventions harnessing its pro-regenerative effects have been examined in the adult mouse spinal cord. Different studies suggest that implanting biomaterials enriched with SHH trophic factors into lesioned spinal cords of adult mice improves recovery and limits scar formation [212, 213]. Thus, it would be beneficial to test the contribution of SHH signaling to innate spinal cord regeneration at neonatal stages.

### Wnt/β-Catenin signaling

Following stab wound injury to the zebrafish telencephalon, Demirci and collaborators observed induction of Wnt/β -catenin signaling components at 20 h post-injury, with their expression returning to baseline levels by 36 h post-injury. Differential gene expression analyses of injured and uninjured brains at various time points suggested that WNT/β-catenin signaling controls expression of several key genes including *p53*, mitogen activated protein kinase (*mapk*), *mTor*, and forkhead box O (*foxo*). This pathway also appears to play a role in regulating apoptosis at early stages of recovery post-injury [69]. The authors suggested that WNT/β-catenin signaling components may play a role in the regulation of cellular and molecular events taking place during early regeneration in the telencephalon. This hypothesis is further supported by studies in the optic tectum showing that WNT regulates ependymo-glia proliferation and differentiation into neurons after injury [70].

Bioinformatic analyses have also identified upregulation of WNT signaling components following zebrafish SCI [67, 97, 214]. Studies in larvae revealed that the pathway is active in ERGs after injury, while they undergo neuronal differentiation. The pathway also enhances axonal regrowth and functional recovery [88]. Further investigation by Wehner et al. employed transgenic reporter lines to visualize the dynamics of WNT-signaling components. The authors found that WNT/β-catenin activation occurs in fibroblast-like cells populating the lesion environment and aligning closely to regenerating axons. WNT directly regulates the transcription of collagen type XII gene (*col12a1a/b*), promoting collagen XII deposition in spinal cord lesion sites. Pharmacological WNT inhibition by IWR- 1 treatment decreased *col12a1a/b* gene expression, which resulted in reduced axonal bridging and swimming ability [76]. This role of WNT during regeneration is conserved in adult zebrafish [98].

Studies investigating WNT signaling in neonatal mice strongly differ in methodology, limiting the scope of cross-species comparison. In vitro studies have demonstrated that neonatal hippocampal NSCs subjected to hypoxic conditions, mimicking brain damage, show upregulation of β-Catenin (*Ctnnb1*), p-GSK-3β (*Gsk3b*), and CyclinD1 (*Ccnd1*), all major WNT pathway effectors [125]. This was associated with increased proliferation and reduced NSC apoptosis. Later in vivo studies suggested that WNT signaling plays a role in guiding neonatal NSC differentiation toward cells with neuronal features after injury [215], indicating a conserved neurogenic function for WNT signaling across species. In agreement with that, a recent study demonstrated activation of WNT/b-Catenin signaling in V-SVZ NSCs and glutamatergic progenitors of neonatal mice exposed to chronic hypoxia [124]. The role of WNT signaling in neonatal spinal cord regeneration is unknown. However, it has been extensively studied in the context of adult SCI and proposed to be involved in the regulation of axon regeneration, neuroinflammation, and remyelination through various mechanisms [216].

### Bone morphogenic protein signaling

Bone morphogenic proteins (BMP) are members of the transforming growth factor beta (TGF-β) super-family, shown to play several crucial roles in neural development and repair [228].

Signaling via BMP may influence long-term recovery from substantial brain damage through regulation of inhibitor of the DNA binding 1 gene *(id1*). Found in RGCs, this gene is highly conserved between zebrafish and mammals, to the extent that human *ID1* gene expression is sufficient to drive NSC generation in zebrafish. Zang et al. demonstrated that BMP/ID1 signaling regulates long-term regenerative capacity following repeated stab wound injuries to the zebrafish telencephalon. While BMPs are expressed by neurons after injury, ID1 is found in NSCs. Modulation of BMP signaling in neurons resulted in changes in *id1* gene expression in NSCs, suggesting a crosstalk between the two cell types after injury. The transcription factor HER4.1, from the HES/HER family, appears to mediate this crosstalk to prevent stem cell pool depletion during regeneration [71].

Although there is limited study of BMP signaling in neonatal mouse brain regeneration, it has been suggested as a promising target for white matter protection in perinatal brain injury [217]. Dizon et al. found that hypoxia–ischemia injury in mice at postnatal day 7 induces BMP4 and SMAD 1/5/8, key effectors of BMP signaling. Antagonizing BMP signaling by overexpressing Noggin resulted in an increase of oligodendrocyte progenitors and oligodendrocytes at 7 days post-injury, along with increased expression of myelin proteins, leading to improved locomotor function at 14 days post-injury [126, 127]. Contribution of BMP to innate spinal cord regeneration in neonatal mice has not been deeply investigated. Parikh et al. showed that SMAD1-dependent BMP signaling is developmentally regulated in mice, and its downregulation with aging contributes to the age-related decline in axon growth potential after SCI [218]. Most recently, BMP signaling has also been shown to act as a negative regulator of scarring by inhibiting collagen deposition after SCI [219].

### Heparin-binding epidermal growth factor signaling

Heparin-binding epidermal growth factor (HB-EGF) is a member of the epidermal growth factor family and serves as a ligand for the receptor tyrosine-protein kinases ERBB1 (EGFR) and ERBB4. Described as a mitogenic and chemoattractive factor, it is initially synthesized as a membrane-bound precursor (pro-HB-EGF) and is later cleaved to form its soluble, secreted form (soluble HB-EGF) [229].

In zebrafish, the HB-EGFa paralogue is significantly upregulated in ERGs lining the spinal cord central canal at 7 days post-injury [86]. The receptors for HB-EGF, ERBB4 and EGFR, which are undetectable in uninjured zebrafish spinal cords, are also highly expressed near the injury site after a transection injury. Animals with *hb-egfa* gene mutations display impairments in swim ability, axon growth, and tissue bridging after spinal cord transection, associated with disrupted indicators of neuron production. Conversely, local recombinant human HB-EGF delivery to spinal cord lesions enhances functional regeneration [86]. In agreement with these findings, HB-EGFa has also been shown to play a role in zebrafish retina and olfactory epithelium neurogenesis [220, 221].

In neonatal mice, HB-EGF has also been implicated in CNS regeneration. Specifically, Scafidi et al. showed that administration of intranasal-HB-EGF reduced apoptosis of myelinating oligodendrocytes preserving axonal myelination and improving behavioral recovery after neonatal hypoxia [128]. In the neonatal mouse spinal cord, adeno-associated virus-mediated overexpression of the gene coding for the human HB-EGF at the lesion site increased the density of serotonergic axon fibers in regions caudal to the injury site [86]. The relevance of HB-EGF for adult mouse spinal cord regeneration remains unclear.

### Extracellular matrix-related signaling

To comprehensively understand species-specific differences in how the brain and spinal cord respond to injury, it is critical to contextualize the restorative mechanisms within the lesion site itself. Research has increasingly focused on the role of the extracellular matrix (ECM) in mediating regeneration. One of the first studies to examine this connection identified fibroblast-derived Collagen XII (COLXII) as a major regulator of axon regeneration in zebrafish [76]. Additionally, in a 2021 study, Tsata and collaborators used both platelet-derived growth factor beta *(Tg(pdgfrb:GFP)* and Cre-based transgenic zebrafish lines to study the role of fibroblasts during spinal cord regeneration. Their finding revealed that fibroblasts, originating from myoseptal and perivascular sources, accumulate at the lesion site where they make direct contact with axonal fascicles extending towards - but not yet crossing - the lesion site. This study highlighted that platelet-derived growth factor (PDGF) signaling in fibroblasts increases expression of axon growth-promoting ECM genes (*cthrc1a* and *col12a1a/b*) while simultaneously decreasing expression of those coding for matrix molecules that hinder regeneration (*lum* and *mfap2*). Genetic ablation of PDGFR-expressing fibroblasts led to reduced axonal bridging after SCI, leading to impaired functional recovery [99]. Further supporting the importance of ECM components, a recent study from Kolb et al. investigated the expression of ECM-derived small leucine-rich proteoglycans (SLRPs) in regenerating zebrafish spinal cords. Typically associated with scar formation in adult mammals (e.g., mice, rats, and humans), SLRPs were found to be largely absent in the regenerating larval zebrafish spinal cord, with the exception of Asporin [77]. When SLRPs were selectively upregulated in the transected zebrafish spinal cord using a *pdgfrb*:SLRP transgenic line, axonal bridge thickness and functional recovery were reduced, suggesting that SLRPs may act as inhibitors of spinal cord regeneration and could be targeted therapeutically to promote repair [77].

The exact role of Collagen XII, PDGF signaling, and SLRP molecules during neonatal mouse CNS regeneration is unknown. A study from Shen et al. suggests that *Pdgfrb* gene deletion disrupts glial scar formation following cerebral ischemia [129]. As for SLRPs, while prominent immunoreactivity has been observed in spinal cord stumps and the lesion core of adult mice [77], expression in neonatal mouse spinal cords awaits investigation. Notably, a study by Li et al. found that expression of ECM genes - such as fibronectin 1 (*Fn1*) and thrombospondin 1 (*Thbs1*) - and their associated regulatory networks, was transiently upregulated in neonatal microglia following a crush injury at postnatal day 2. The deposition of these ECM molecules contributes to the growth-conducive environment seen in neonatal mice and is one of the key factors differentiating the regenerative responses of neonates versus adults after SCI [113].

### Tissue regeneration enhancer elements

Recent studies have identified tissue regeneration enhancer elements (TREEs) and silencer elements (TRSEs) as key regulators of regeneration in several zebrafish tissues [230–233]. Epigenetic analysis of injured zebrafish spinal cords led to the discovery of several putative regions activating regeneration associated expression in the regenerating spinal cord. Among them, regions associated with the genes coding for the heparin-binding epidermal growth factor a (*hb-egfa)*, the nuclear receptorcoactivator 4 *(ncoa 4)*,the mRNA-processing factor 38fb *(prp38fb)*, and the suppressor of hairless 2 *(ssuh2)* were experimentally validated in transgenic enhancer reporter lines [86]. Additionally, a cis-regulatory element necessary to direct expression of *ccn2a*, which codes for the bridging glial marker CTGFa, after SCI has also been reported [94].

Neonatal mouse-specific spinal cord regeneration enhancers have not yet been investigated. Shu et al. in a recent study examined chromatin accessibility profiles at the single-cell level in mouse neural tubes from embryonic days 9.5 to 13.5. The authors identified specific cis-regulatory elements in neural progenitors and neurons, highlighting enhancer networks as a general mechanism in transcriptional regulation during development [234]. Further investigations into the chromatin structure and transcription complexes in mammalian spinal cord tissue at different stages, along with their ability to recognize additional TREEs, are likely to offer valuable insights into the decline of regenerative capacity with age. Strikingly, when certain zebrafish spinal cord enhancers (i.e., *hb-egfa*EN) were delivered to neonatal mice using adenoviral vectors, they successfully directed gene expression at injury sites in neonatal mice, which can regenerate axons, similar to zebrafish. However, this response did not occur in adult mice, where regeneration is limited. This suggests that the transcriptional machinery and/or the injury-induced transcription chromatin status of regenerating zebrafish and neonatal mice share similarities that are possibly lost with aging [86]. This cross-species enhancer recognition makes chromatin regulation an intriguing target for future translational study in CNS regeneration and plasticity [235, 236].

## Conserved immune cell contributions to CNS repair

Immune cells play pivotal roles in CNS regeneration. In both zebrafish and mice, injury leads to activation and recruitment of resident microglia, peripheral neutrophils, macrophages, and other leukocytes and lymphocytes to the injury site. The ability of these cells to either promote or inhibit healing is determined by their subpopulation composition and activation kinetics. When immune cells function optimally, they clear debris, protect healthy tissue, and promote healing. Conversely, unresolved or excessive inflammation can lead to impaired regeneration and scar formation. While our understanding of these intricate cellular dynamics has notably evolved over the last decade, an incomplete characterization of immune cells’ multifaceted, transient roles in CNS regeneration limits cross-species comparison. For instance, adaptive immunity is becoming increasingly recognized as a contributor to regeneration outcomes in zebrafish but lacks similar attention in mammalian neonatal models of CNS injury. T-regulatory (T_reg_) cells accumulate at the lesion site in zebrafish spinal cords between 3 and 7-day post injury. More specifically, FOXP3a-expressing T cells, also known as zT_reg_ cells, produce local trophic factors such as neurotrophin- 3 that facilitate ERG proliferation after injury. Ablating zT_reg_ cells from zebrafish with SCI resulted in disorganized rostral and caudal axonal sprouting at 30 days post-injury, impaired functional recovery, and decreased expression of key neurogenic factor genes such as *gdnfa* and *ngfb* at 7 days post-injury [100]. Though similar studies have not been performed in neonatal mice, observations in adult mice with SCI revealed that T_reg_ cells accumulate at the lesion [237]. Controlled reduction of T_reg_ levels early after injury positively influenced the repair process; however, their ablation during the subacute or chronic phase disrupted tissue remodeling [237]. These findings emphasize the crucial spatial and temporal dynamics of effector and regulatory T cells, with their balance playing a key role in the CNS repair process. In the following sections, we discuss immune cell populations that have been implicated and well documented in pro-regenerative zebrafish and neonatal mouse responses to CNS damage (Fig. 3, Table 2).

### Neutrophils

Precise intercellular signaling is required for CNS regeneration in zebrafish and mice. Palsamy et al. [72] employed a telencephalic injury model in microglia-depleted adult zebrafish, observing a compensatory accumulation of neutrophils between 2 and 4 days post-injury, leading to a prolonged inflammatory phase. Efficient clearance of neutrophils is a critical step for tissue repair in the zebrafish CNS, and macrophages are necessary for controlling neutrophil levels during later stages of repair [78]. In a recent study, de Sena-Tomás et al. demonstrated that neutrophils are recruited to the larval spinal cord lesion site to then reverse migrate throughout the body. Promoting neutrophil inflammation resolution by inhibiting C-X-C chemokine receptor type 4 (CXCR4) signaling boosts cellular and functional regeneration [79]. Similar observations were also reported in neonatal mice in a recent study from Kitade et al. Specifically, flow cytometry analysis of crush injured spinal cords showed that neonatal astrocytes secrete lower levels of chemokines (such as C-X-C motif chemokine ligand 1 and 2, CXCL1 and CXCL2) to recruit circulating neutrophils after SCI compared to adult astrocytes. Neonatal circulating neutrophils also expressed lower levels of the chemokine receptor CXCR2 and adhesion molecule integrin β2 compared to adults. This resulted in reduced neutrophil recruitment and lower levels of inflammatory cytokines at the injury site, leading to fewer apoptotic neurons, improved axonal regeneration, and better locomotor recovery than adults [114]. These findings suggest that limiting neutrophil infiltration may enhance regeneration.

### Blood-derived macrophages

In zebrafish, macrophages regulate axon regeneration by producing tumor necrosis factor alpha (TNF-α) after SCI and reducing interleukin 1 beta (IL 1β) levels [78]. TNF-α induces TNFRSF1A-mediated AP-1 activity in ERGs to increase regeneration-promoting expression of histone deacetylase 1 gene (*hdac1*) and neurogenesis. This suggests important macrophage crosstalk with spinal ERG progenitors after injury [80]. Macrophages are also thought to be involved in promoting expression of additional pro-regenerative genes in zebrafish, such as *tgfb1a* and *tgfb3*, and to control concentrations of neutrophil-derived pro-inflammatory cytokines following SCI [81].

Comparing these results to observations in mice suggests that the regenerative role of macrophages in response to SCI is dependent on their eventual withdrawal from the lesion site. In the spinal cord of injured neonatal and adult mice, macrophages were pervasive at 3 days post-injury. Interestingly, at 14 days post-injury, macrophage accumulation persisted in the adult lesion but were absent from the neonatal lesion [113]. Macrophages have been implicated in age-dependent control of profibrotic myelin-derived cholesterol at the lesion site. Specifically, Zheng and collogues showed that after SCI in adult mice, myelin-derived cholesterol crystals are deposited at the lesion site and engulphed by macrophages. This process perpetuates the macrophage inflammatory state and promotes scar formation. By contrast, in neonatal mice, the macrophage population present at 3 days post injury is resolved within 2 weeks, as are cholesterol crystals, suggesting that effective, homeostatic cholesterol transport and macrophage clearance are lost with age. When macrophages overloaded with myelin-derived cholesterol were injected into the neonatal lesion site, scar formation occurred, indicating that excess cholesterol accumulation exacerbates macrophage activation and impairs healing [223]. While literature on the role of macrophages in brain regeneration in neonatal mice is limited, a closer examination of macrophage subtypes, functions, and, activation dynamics after injury may reveal a more nuanced understanding of species-specific differences in macrophage activity.

### Microglia

It is widely known that microglia are heavily involved in innate inflammatory responses to injury. Under physiological conditions, a “resting” microglial cell is characterized by a very small cell body with elongated, ramified processes [238]. Microglia produce neurotrophic and anti-inflammatory factors to support the normal function of neurons and glial cells [239]. Demonstrating striking plasticity, microglia can quickly react to any sign of tissue damage by secreting both pro- and anti-inflammatory molecules at different stages of repair after neural trauma [240, 241].

In the adult zebrafish brain, microglia proliferate to populate the lesion site for many days following injury, to effectively clear debris and activate neurogenesis (reviewed in [222]). Return of microglia to their basal state is a granulin-dependent process [73]. Supporting the requirement for microglia to achieve regeneration, pharmacological and genetic inactivation of microglia after telencephalic injury led to a persistent lesion, a prolonged inflammatory response, and reduced pro-regenerative signaling, despite the absence of glial scar formation [72]. One could speculate that the absence of scarring in zebrafish could be due to the lack of free astrocytes in their CNS. However, adult ERGs in the brain and spinal cord fulfill astrocytic functions. Also, astrocyte-like cells have been reported in the developing zebrafish brain, although their existence is still debated [242].

Microglia also play a major role in zebrafish spinal cord regeneration. In the spinal cord of zebrafish larvae, targeted chemogenetic ablation of neurons activates microglia, resulting in microglia’s phagocytic ingestion of neuronal remnants within 20–30 minutes. This response is diminished when larvae are exposed to the immunosuppressant dexamethasone, a condition that impairs motor neuron regeneration [108]. In an additional larval study, Tsarouchas et al. analyzed the requirement for microglia during regeneration using *csf1ra/b* zebrafish mutants, in which the function of colony stimulating factor 1 receptor (CSF1R), needed for microglia differentiation, is compromised. Axon regeneration was unaffected in these mutants compared to wild types. However, the quantity of peripheral macrophages responding to injury increased in *csf1ra/b* mutant larvae, likely compensating for a possible regeneration promoting role of microglia [78].

In line with what is observed in zebrafish, microglial cells are crucial for brain and spinal cord regeneration in neonatal mice. In the neonatal mouse brain, microglial cells are key players in the innate inflammatory response and help limit secondary damage, such as hemorrhage [224]. In a hypoxic-ischemic brain injury model, microglia-depleted male mice exhibited larger infarct volumes compared to microglia-depleted females, who instead showed a greater number of apoptotic neurons in the hippocampus and the thalamus. These findings suggest that microglial cells are required to contain damage, with sex-based differences, as males were more affected than females. Interestingly, microglia depletion in both sexes also led to reduced IL-6 and TGF-β levels [130]. Injury in this study occurred at postnatal day 10, which falls beyond the critical neonatal regeneration window of postnatal day 0 and 8. In a differently designed study, Bourget et al. inactivated microglia using metformin or induced its chemical ablation in mice undergoing hypoxia–ischemia at postnatal day 8, within the first week post damage. This led to improved behavioral outcomes indicating that dampening or ablating the early microglia response is sufficient to protect against functional deficits [131]. The conflicting data regarding the impact of microglia on fine motor and cognitive behavior after neonatal brain injury warrants further research.

In the neonatal mouse spinal cord, injury results in the appearance of five transcriptionally distinct microglial clusters [113]. One of these clusters, located around the lesion site, transiently expresses high levels of fibronectin 1 (FN1), along with proteinase inhibitors, possibly resolving inflammation at 3 days post-injury, with expression ceasing by 5 days post injury. Microglial-derived FN1 has been proposed to form a bridge across the lesion site that supports growth of regenerating axons. Unlike neonatal microglia, adult mouse microglia show no significant induction of *Fn1* gene and proteinase inhibitor levels after injury, which might contribute to the lack of regenerative capacity in the adult animal. In support of the potentially regenerating role of microglial-derived FN1 and protease inhibitors, treatment of adult microglia with two chemical proteinase inhibitors—E64, a membrane-permeable irreversible inhibitor of a broad range of cysteine peptidases, and serpina3n, a serine protease inhibitor—followed by transplantation into adult spinal cord lesions, improved axon regeneration in adult mice [113].

## Conclusion and perspectives

Since the time of Cajal, the brain and spinal cord of humans and other adult mammals have been broadly regarded as organs with little to no capacity for regeneration. It is only within the past four decades that researchers have identified the capacity of the nervous system to change in response to damage or experience [243–246]. Despite remarkable scientific advancement during this time, no cure currently exists for spinal cord or traumatic brain injuries. Recent studies in zebrafish and neonatal murine models are providing valuable insights to bolster regenerative potential in adult mammals. Notably, comparative studies have revealed that some pro-regenerative pathways and mechanisms might be conserved between zebrafish and neonatal mice but may diminish or change as mammals age, possibly taking on different roles. The guiding principles behind this variation are still a mystery. Differences in tissue complexity, in mechanisms enhancing and silencing gene networks in response to injury, in metabolic demands and in organism size and reproductive strategies, may all play a role in favoring repair in zebrafish and neonatal mice and/or opposing it in adult mice. The integration of a diverse number of contextual cues, both internal and external, may also influence the way that an organism responds to CNS injury, but more importantly how injury signals are received and transmitted to surrounding cells. Considering these aspects across species will foster the translation of basic science into clinically useful treatments for the broad group of patients who suffer from CNS disorders.

More research is needed to uncover why regeneration declines in adult mammals and how it might be reactivated. For example, identifying pro-regenerative cell subsets with specific molecular markers in neonatal mice would enable researchers to track the fate of these cells as they mature into adulthood. This would provide valuable insights into their role in tissue repair and regeneration over time and help determine whether these cells retain their regenerative potential or if they can be reactivated. Additionally, investigating how age-related changes in chromatin modifications affect tissue regeneration could provide crucial information. Advances in research tools and imaging techniques will help explore these processes in greater detail. Although still beyond immediate reach, the reactivation of regeneration in adult mammals—and perhaps humans—is a realistic prospect and should encourage scientific research, potentially leading to transformative discovery and innovative treatments for CNS disorders.

## Data Availability

No datasets were generated or analysed during the current study.

## Abbreviations

5-HT1B: 5-Hydroxytryptamine receptor 1B
*Ascl1*: Achaete-scute family BHLH transcription factor 1 gene
*Atoh1*: Atonal BHLH (Basic helix–loop–helix) transcription factor 1 gene
ATTAC: Apoptosis through targeted activation of caspase 8
BHLH: Basic helix–loop–helix
BLBP: Brain lipid-binding protein
BMP: Bone morphogenetic protein
BMP4: Bone morphogenetic protein 4
BrdU: Bromodeoxyuridine
*Cacna2d2*: Calcium voltage-gated channel auxiliary subunit alpha2 delta 2 gene
CALB1: Calbindin 1
CC: Central canal
*ccn2a*: Cellular communication network factor 2a
*Ccnd1*: Cyclin D1 gene
CNS: Central nervous system
COLXII: Collagen XII
*col12a1a/b*: Collagen type XII gene
CRISPR: Clustered regularly interspaced short palindromic repeats
CSF: Cerebrospinal fluid
CSF1R: Colony-stimulating factor 1 receptor
*Csf1ra/b*: Colony-stimulating factor 1 receptor a/b genes
CTGFa: Connective tissue growth factor a
*cthrc1a*: Collagen triple helix repeat containing 1a gene
*Ctnnb1*: B-Catenin 1
CXCL1/CXCL2: C-X-C Motif Chemokine Ligand 1 and 2
CXCR4: C-X-C chemokine receptor type 4
Dl: Dorsal telencephalon lateral zone
DNA: Deoxyribonucleic acid
Dp: Dorsal telencephalon posterior zone
ECM: Extracellular matrix
EGFR: Epidermal growth factor receptor
ERBB1: Receptor Tyrosine Kinase erbB- 1
ERBB4: Receptor Tyrosine Kinase erbB- 4
ERG: Ependymo-radial glia
EZH2 *(Ezh2)*: Histone methyltransferase enhancer of zeste homolog 2
FN1 (*Fn1*): Fibronectin 1
FOXP2: Forkhead box protein 2
FOXJ1: Forkhead box j1
FOXJ1a: Forkhead box j1 paralogue a
*foxo*: Forkhead box O
GCL: Granular cell layer
*gdnfa*: Glial cell line-derived neurotrophic factor
GFAP: Glial fibrillary acidic protein
GFP: Green fluorescent protein
GS: Glutamine synthetase
*Gsk3b*: Glygcogen synthase kinase- 3b gene
HB-EGF: Heparin-binding epidermal growth factor
*hb-egfa*: Heparin-binding epidermal growth factor paralogue a gene
*hb-egfa*EN: Heparin-binding epidermal growth factor paralogue a enhancer
*hdac1*: Histone deacetylase 1
HER4: Hairy-related 4
HEY1: HES-related family BHLH transcription factor with YRPW motif 1
HH: Hedgehog
ID1 (*id1*): DNA binding 1
IL- 1β: Interleukin- 1 beta
IL- 6: Interleukin- 6
KLF4: Krüppel-like factor 4
*lum*: Lumican gene
LV: Lateral ventricle
*mapk*: Mitogen activated protein kinase gene
*mfap2*: Microfibrillar-associated protein 2 gene
ML: Molecular layer
MSX2: Msh homebox 2
mTOR (*mTor*): Mammalian 391 target of rapamycin
*ncoa4*: Nuclear receptor coactivator 4
*ngfb*: Nerve growth factor beta polypeptide gene
NPC: Neural progenitor cell
NSC: Neural stem cell
OCT4: Octamer-binding transcription factor 4
OLIG2: Oligodendrocyte transcription factor 2
PAX6: Paired box protein 6
PCNA: Proliferating cell nuclear antigen
PDGFR: Platelet-derived growth factor
*pdgfrb*: Platelet-derived growth factor beta gene
PLX 3397: Pexidartinib
*prp38fb*: Pre-mRNA processing factor 38B gene
PSA-NCAM: Polysialylated neuronal cell adhesion molecule
PTEN (*pten*): Phosphatase and tensin homolog
PTENa/PTENb: Phosphatase and tensin homolog a/phosphatase and tensin homolog b
*ptf1a*: Pancreas-associated transcription factor 1a gene
RGC: Radial glial cell
RMS: Rostral migratory stream
SCI: Spinal cord injury
SGZ: Sub-granular zone
SHH (*shh)*: Sonic hedgehog
SHHa/SHHb: Sonic hedgehog ligand a/sonic hedgehog ligand b
SIRT1: Sirtuin 1
SLRP: Small leucine-rich proteoglycan
SMAD1/5/8: Mothers against decapentaplegic homolog 1/5/8
SOCS3: Suppressor of cytokine signaling 3
SOX2: SRY-Box 2
SOX10: SRY-Box 10
TRSEs: Tissue regeneration silencer elements
*ssuh2*: Ssu- 2 Homolog
TAZ (Taz): Transcriptional co-activator with PDZ-binding motif
TBI: Traumatic brain injury
TEAD: Transcriptional enhanced associate domain
TelV: Telencephalic ventricle
TET1/TET2: Ten-eleven translocation methylcytosine dioxygenase 1 and 2 1
TGF-β: Transforming growth factor beta
*tgfb1a/tgfb3*: Transforming growth factor beta 1a and 3
*Thbs1*: Thrombospondin 1
TNF-α: Tumor necrosis factor alpha
TNFRSF1A: TNF receptor superfamily member 1 A
TREEs: Tissue regeneration enhancer elements
T_reg_: T-regulatory
*twist1*: Twist-related protein 1
*twist1a*: Twist-related protein 1a
V-SVZ: Ventricular-subventricular zone
Vd: Dorsal nucleus of ventral telencephalonic area
Vv: Ventral nucleus of ventral telencephalonic area
VZ: Ventricular zone
WNT (Wnt): Wingless-type MMTV integration site family
YAP: Yes-associated protein
YAP1: Yes-associated protein 1
zT_reg_: Foxp3a-expressing T cells
